# Significance of post‐progression therapy after tyrosine kinase inhibitors for advanced hepatocellular carcinoma

**DOI:** 10.1002/jgh3.12772

**Published:** 2022-05-25

**Authors:** Yoshihiko Yano, Atsushi Yamamoto, Akihiro Minami, Kenji Momose, Takuya Mimura, Soo Ki Kim, Hiroki Hayashi, Takuo Kado, Hirotaka Hirano, Seiya Hirohata, Seitetsu Yoon, Katsuhisa Nishi, Hiroshi Tei, Hidenori Tanaka, Sachiko Oouchi, Takanori Matsuura, Eiichiro Yasutomi, Yuri Hatazawa, Yuuki Shiomi, Yoshihide Ueda, Yuzo Kodama, Kobe Liver Conference (KLC)

**Affiliations:** ^1^ Division of gastroenterology, Department of internal medicine Kobe University Graduate School of Medicine Kobe Japan; ^2^ Department of Gastroenterology Konan Medical Center Kobe Japan; ^3^ Department of Gastroenterology Osaka Saiseikai Nakatsu Hospital Osaka Japan; ^4^ Department of Gastroenterology Hyogo Prefectural Hyogo Cancer Center Kobe Japan; ^5^ Department of internal medicine Kobe Asahi Hospital Kobe Japan; ^6^ Department of Gastroenterology Kitaharima Medical Center Ono Japan; ^7^ Department of Gastroenterology Akashi Medical Center Akashi Japan; ^8^ Department of Gastroenterology Yodogawa Christian Hospital Osaka Japan; ^9^ Department of Gastroenterology Hyogo Prefectural Kakogawa Medical Center Kakogawa Japan; ^10^ Department of Gastroenterology Hyogo Prefectural Awaji Medical Center Sumoto Japan; ^11^ Department of Gastroenterology Kobe City Medical Center General Hospital Kobe Japan; ^12^ Department of Gastroenterology Sanda City Hospital Sanda Japan; ^13^ Steel Memorial Hirohata Hospital Himeji Japan

**Keywords:** hepatocellular carcinoma, post‐progression therapy, tyrosine kinase inhibitor

## Abstract

**Background and Aim:**

Molecular‐targeted therapies such as sorafenib and lenvatinib have long been used as first‐line treatment for advanced hepatocellular carcinoma (aHCC). However, adverse events or limited therapeutic effects may necessitate the change to another therapeutic option, known as post‐progression therapy. To investigate the significance of post‐progression therapy, we analyzed the outcomes of aHCC patients following first‐line molecular‐targeted therapy in a real‐world study.

**Methods:**

This retrospective, multicenter study involved patients with aHCC who received sorafenib or lenvatinib as first‐line therapy between January 2011 and September 2021.

**Results:**

In total, 513 patients were analyzed: 309 treated with sorafenib and 204 with lenvatinib. The overall response and disease control rates were 15 and 50%, respectively, in the sorafenib group and 30 and 75%, respectively, in the lenvatinib group (*P* < 0.001). Kaplan–Meier analysis revealed no significant differences in progression‐free survival and overall survival (OS) between the two treatments. Multivariate analysis revealed that fibrosis‐4 index, disease control rate, post‐progression therapy, and use of an immune checkpoint inhibitor (ICI) were significantly associated with OS. OS was significantly longer in patients who received post‐progression therapy than in those who did not (log‐rank *P* < 0.001). Most patients who received an ICI as post‐progression therapy had previously received lenvatinib. Among lenvatinib‐treated patients, OS was significantly longer in patients who received an ICI than in patients received another or no post‐progression therapy (*P* = 0.004).

**Conclusion:**

The introduction of newer drugs for post‐progression therapy is expected to prolong survival. ICI‐based regimens appear to be effective after lenvatinib.

## Graphical abstract

This is a retrospective multicenter study involving patients with advanced hepatocellular carcinoma who received sorafenib or lenvatinib as first‐line therapy. The study revealed that the presence or absence of post‐progression therapy after tyrosine kinase inhibitor therapy plays a role in overall survival, especially the efficacy of regimens including immune checkpoint inhibitor after lenvatinib treatment.

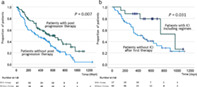

## Introduction

The tyrosine kinase inhibitors (TKIs) sorafenib and lenvatinib are widely used to treat patients with transcatheter arterial chemoembolization (TACE)‐refractory hepatocellular carcinoma (HCC) worldwide. Sorafenib was approved by the US Food and Drug Administration in 2007 as systemic chemotherapy for unresectable advanced HCC (aHCC). In the double‐blind, placebo‐controlled SHARP trial conducted in Europe and the United States, the overall survival (OS) improved from 7.9 months in the placebo group to 10.7 months in the sorafenib group.^1^ In the Asia–Pacific region, the ORIENTAL trial showed a survival benefit, with an OS of 6.5 months in the sorafenib group *versus* 4.2 months in the placebo group.^2^ The phase III REFLECT trial was designed to investigate the non‐inferiority of lenvatinib *versus* sorafenib as first‐line therapy for unresectable HCC. In that trial, the lenvatinib group had non‐inferior progression‐free survival (PFS) of 7.3 months and OS of 13.6 months compared with sorafenib (PFS of 3.7 months and OS of 12.3 months).^3^ Meanwhile, newer agents such as regorafenib, cabozantinib, and ramucirumab have been shown to be effective as second‐line agents after sorafenib, but their efficacy after lenvatinib has not yet been established.^4^, ^5^, ^6^ In 2020, the combination of atezolizumab and bevacizumab was shown to be effective as first‐line therapy for HCC,^7^ but the efficacy of this combination has not been assessed following treatment with a TKI. Therefore, in this observational study, we examined the efficacy of molecular‐targeted drugs for the treatment of HCC in real‐world clinical practice, as well as the efficacy of post‐progression therapy after a TKI, with a focus on the use of immune checkpoint inhibitors (ICIs) for post‐progression therapy.

## Methods

### Patients

A total of 513 patients who received sorafenib or lenvatinib as first‐line therapy between January 2010 and September 2021 at Kobe University Hospital and its affiliated institutions were included in the study. Sorafenib and lenvatinib were introduced for TACE‐refractory HCC or HCC with extrahepatic by the attending physician. The Up‐to‐7 criteria were used for HCCs, with 7 as the sum of the size of the largest tumor (in cm) and the number of tumors.^8^ Radiological assessments were evaluated according to modified Response Evaluation Criteria in Solid Tumors (mRECIST).^9^, ^10^ Adverse events (AEs) were assessed and recorded using the Common Terminology Criteria for Adverse Events version 4.0. Treatment discontinuation of sorafenib and lenvatinib was based on the AEs and assessment of the treatment efficacy. The next treatment after discontinuation of treatment was selected based on the judgment of the attending physician. This study was approved by the Ethics Committee of Kobe University (B200215). The cut‐off date for data collection was 30 September 2021.

### Statistical analysis

Paired *t*‐tests, Kaplan–Meier analysis, and log‐rank tests were used for statistical analyses. The factors were then entered into a stepwise logistic regression model using OS as the dependent variable. In all analyses, *P*‐values of <0.05 were considered statistically significant. All analyses were carried out using SPSS v. 28 (IBM, Armonk, NY, USA).

The study was carried out in accordance with the Declaration of Helsinki and was approved by the institutional review board of the Kobe University Graduate School of Medicine and by the institutional review boards of the participating hospitals (no. B200215).

## Results

### Patient baseline characteristics

The baseline characteristics of 513 patients are summarized in Table 1. Their ages ranged from 29 to 90 years with a mean ± SD of 70.2 ± 9.9 years and a median of 72 years. Sorafenib was administered to 309 patients and lenvatinib to 204 patients. Treatments before sorafenib or lenvatinib were surgery in 202 patients, radiofrequency ablation in 162 patients, TACE in 393 patients, and radiotherapy in 119 patients. The numbers of patients classified as Child–Pugh (CP) A/B were 399/114, and the numbers of patients with clinical stage II/III/IV were 67/145/265, respectively. Overall, 233 (45%) patients had extrahepatic metastases and 107 (21%) had vascular invasion. The numbers of patients within/outside the Up‐to‐7 criteria were 231/362, respectively. The sorafenib group was significantly younger, and viral hepatitis was the predominant etiology compared with the lenvatinib group. The frequency of diabetes, on the other hand, was significantly higher in the lenvatinib group. There were no significant differences in the markers of liver function, such as the fibrosis‐4 (FIB‐4) index, CP score, and the albumin–bilirubin (ALBI) grade. However, the tumor‐node‐metastasis (TNM) stage, the frequency of extrahepatic metastasis, and the frequency of being within the Up‐to‐7 criteria were significantly greater in the sorafenib group (Table 1).

**Table 1:** Characteristics of patients with advanced hepatocellular carcinoma

	All	Sorafenib	Lenvatinib	*P*‐value
*N*	513	309	204	
Age (years)	70.0 ± 10.0	69.0 ± 10.0	71.9 ± 9.6	0.001
Sex (male/female)	426/87	261/48	165/39	NS
BMI (kg/m^2^)	23.3 ± 4.5	23.4 ± 5.3	23.5 ± 3.7	NS
Etiology (viral/non‐viral)	312/201	207/102	106/98	<0.001
Complication: hypertension	282 (55%)	161 (52%)	121 (59%)	NS
Complication: diabetes	154 (30%)	88 (28%)	66 (32%)	NS
Pretreatment (TACE/OPE/RFA)	393/202/162	242/134/99	151/68/63	NS
PLT (×10^3^/mm^4^)	14.7 ± 7.7	14.1 ± 7.2	15.7 ± 8.4	0.025
AST (IU/L)	51.1 ± 39.2	52.7 ± 34.6	48.6 ± 45.5	NS
ALT (IU/L)	36.1 ± 26.9	37.9 ± 26.3	33.5 ± 27.6	NS
Total bilirubin (mg/dL)	0.93 ± 0.53	0.94 ± 0.57	0.91 ± 0.47	NS
Albumin (g/dL)	3.61 ± 0.54	3.63 ± 0.54	3.58 ± 0.53	NS
Prothrombin time (INR)	1.09 ± 0.14	1.09 ± 0.13	1.09 ± 0.15	NS
AFP (ng/mL) (>400)	180 (35%)	117 (38%)	63 (31%)	NS
AFP (ng/mL) (median)	64.0	133.7	28.1	NS
DCP (mAU/mL) (>400)	262 (51%)	170 (55%)	92 (45%)	NS
DCP (mAU/mL) (median)	421.0	608.0	246.0	NS
APRI	1.12 ± 1.03	1.20 ± 1.04	1.00 ± 0.99	NS
FIB‐4 index	5.26 ± 4.43	5.25 ± 3.68	5.29 ± 5.42	NS
ECOG PS (0/1/2)	312/104/17	200/45/4	122/59/13	NS
Child–Pugh grade (A/B)	399/114	253/56	146/58	NS
Child–Pugh score	6.04 ± 1.23	6.01 ± 1.20	6.07 ± 1.27	NS
ALBI grade (1/2/3)	166/320/27	107/187/15	59/133/12	NS
ALBI score	−1.73 ± 0.55	−1.70 ± 0.55	−1.76 ± 0.54	NS
TNM stage (2/3/4)	67/145/265	20/78/188	47/67/77	<0.01
Vessel invasion	107 (21%)	62 (20%)	45 (22%)	NS
Extrahepatic metastasis	233 (45%)	160 (52%)	73 (35%)	<0.001
Within Up‐to‐7 criteria	231 (45%)	155 (50%)	75 (37%)	0.007

Values are *n*, *n* (%), or mean ± SD, unless otherwise specified.

AFP, alpha‐fetoprotein; ALBI, albumin–bilirubin; ALT, alanine aminotransferase; APRI, AST‐to‐platelet ratio index; AST, aspartate aminotransferase; BMI, body mass index; DCP, des‐gamma‐carboxy pro‐ thrombin; ECOG PS, Eastern Cooperative Oncology Group performance status; FIB‐4, fibrosis‐4; INR, international normalized ratio; NS, not significant; OPE, operation; PLT, platelet; RFA, radiofrequency ablation; TACE, transcatheter arterial chemoembolization.

### Treatment efficacy and AEs

Table 2 summarizes the therapeutic efficacy and the degrees of the five most frequent AEs.

**Table 2:** Efficacy and adverse effects of treatment

	All	Sorafenib	Lenvatinib	*P*‐value
*N*	513	309	204	
Duration of treatment	111 (1–1932)	107 (1–1932)	115 (1–922)	NS
ORR	21%	15%	30%	<0.001
DCR	59%	50%	75%	<0.001
AE, total	64%	64%	65%	NS
AE, HFS	24%	28%	18%	0.002
AE, proteinuria	7%	4%	12%	0.004
AE, hypertension	16%	14%	18%	NS
AE, fatigue	13%	11%	15%	NS
AE, appetite loss	9%	7%	11%	NS

Values are median (range) or percent of patients.

AE, adverse effect; DCR, disease control rate; HFS, hand foot syndrome; NS, not significant; ORR, overall response rate; OS, overall survival; PFS, progression‐free survival.

**Figure 1:**
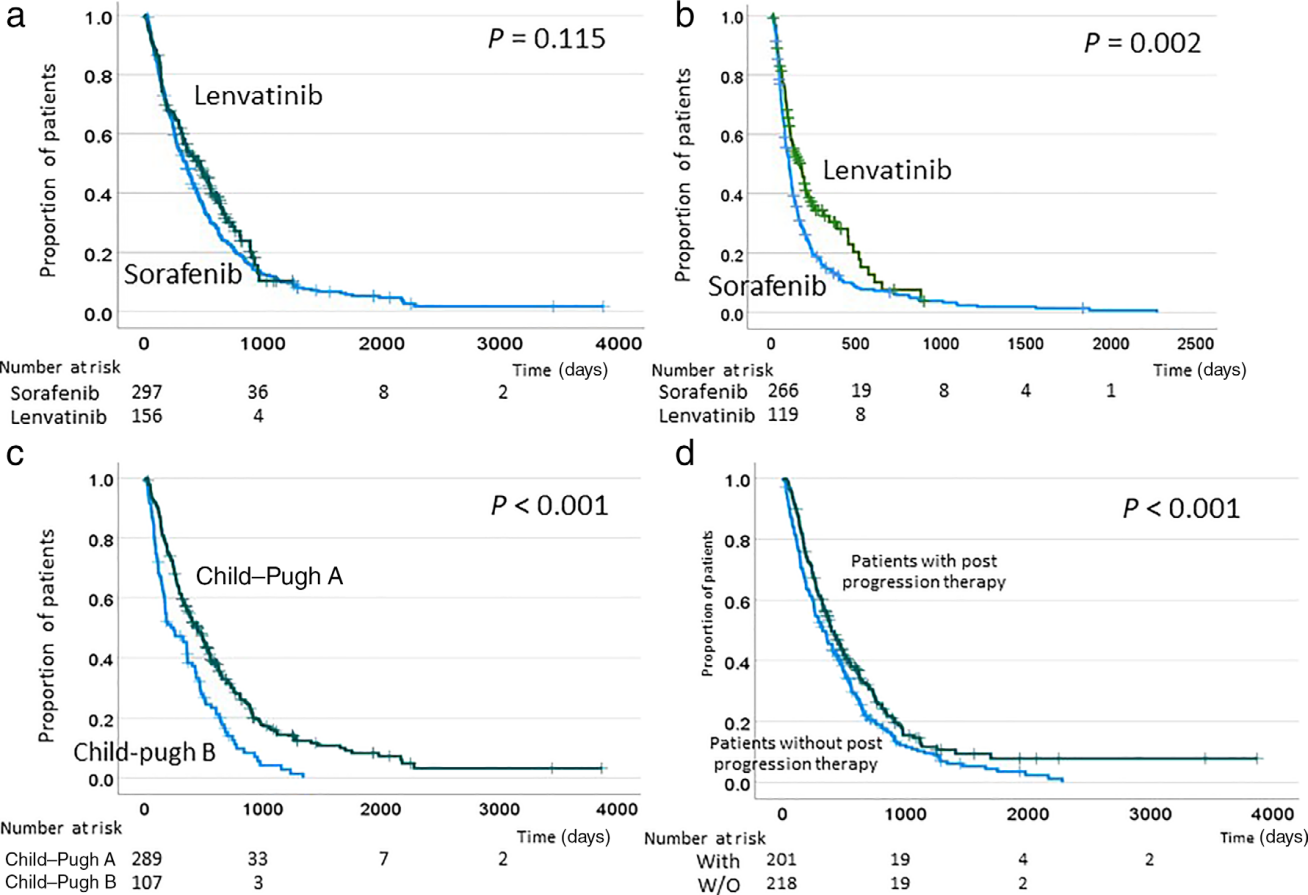
Kaplan–Meier plots of (a) overall survival and (b) progression‐free survival according to treatment with sorafenib and lenvatinib. Kaplan–Meier plot of (c) overall survival according to the Child–Pugh grade and (d) use of post‐progression therapy. With: patients who received post‐progression therapy; W/O: patients who did not receive post‐progression therapy.

The duration of treatment ranged from 1 to 1932 days (median 107 days) in the sorafenib group and from 1 to 922 days (median 115 days) in the lenvatinib group, which were not significantly different. According to the log‐rank test, PFS of lenvatinib group was significantly longer than that of sorafenib group, although OS was not significantly different between the two groups (Fig. 1a,b). In addition, OS was significantly longer in patients with CP score A compared to those with CP score B (Fig. 1c). To reduce confounding effects, propensity score matching analysis was performed between the sorafenib and lenvatinib groups. Factors including age, etiology, CP score, frequency of extrahepatic metastasis, and frequency within Up‐to‐7 criteria were adjusted, and the analysis included 163 patients for each of sorafenib and lenvatinib. Finally, OS and PFS were not significantly different between the two matched groups (Figure S1).

### Factors associated with OS

In the univariate analyses, the FIB‐4 index before treatment, disease control rate, the availability of post‐progression therapy, and the use of an ICI as post‐progression therapy were significantly associated with OS (Table 3). Subsequently, factors that were statistically significant (*P* < 0.05) in the univariate analysis were subjected to multivariate analysis, which also revealed that the four factors were significantly associated with OS. In total, 226 patients received post‐progression therapy after first‐line TKI therapy (Table 4). Log‐rank analysis demonstrated a significant increase in OS in patients who received post‐progression therapy than in patients who did not (Fig. 1d).

**Table 3:** Univariate and multivariate analyses of factors associated with overall survival

	Univariate analysis	Multivariate analysis		
	*P*‐value	HR	95% CI	*P*‐value
Age, 75 years	0.554			
Sex, male	0.852			
Etiology, viral	0.191			
Therapy, lenvatinib	0.223			
ECOG PS, >0	0.192			
AFP, >400	0.212			
DCP, >400	0.116			
APRI, >1.00	0.904			
FIB‐4 index, >2.67	0.013	8.975	1.81–44.51	0.007
Major vessel invasion	0.712			
Extrahepatic spread	0.631			
Child–Pugh, >B	0.192			
ALBI, >1	0.173			
Up‐to‐7, within	0.124			
ORR	0.246			
DCR	<0.001	11.805	2.57–44.80	0.001
Post‐progression therapy	<0.001	7.811	2.02–30.16	0.003
TKI included in post‐progression therapy	0.611			
ICI included in post‐progression therapy	0.045	16.55	1.30–211.36	0.031
AE, grade > 2	0.204			

AE, adverse event; AFP, alpha‐fetoprotein; ALBI, albumin‐bilirubin; APRI, aspartate aminotransferase to platelet ratio index; CI, confidence interval; DCP, des‐gamma‐carboxy pro‐thrombin; DCR, disease control rate; FIB‐4, fibrosis‐4; HR, hazard ratio; ICI, immune checkpoint inhibitor; ORR, overall response rate; TKI, tyrosine kinase inhibitor.

**Table 4:** Characteristics of patients with and without post‐progression therapy at the first‐line tyrosine kinase inhibitor therapy

	With post‐progression therapy	Without post‐progression therapy	*P*‐value
*N*	226	287	
Age (years)	70.2 ± 8.9	69.8 ± 10.9	NS
Sex (male/female)	192/34	232/55	NS
BMI (kg/m^2^)	23.4 ± 3.7	23.4 ± 5.6	NS
Etiology (viral/non‐viral)	136/90	169/118	NS
Complication: hypertension	131 (58%)	149 (52%)	NS
Complication: diabetes	63 (28%)	92 (32%)	NS
AFP (ng/mL) (>400)	73 (32%)	108 (38%)	NS
DCP (mAU/mL) (>400)	117 (52%)	175 (50%)	NS
APRI	1.05 ± 0.97	1.17 ± 1.04	0.031
FIB‐4 index	4.97 ± 0.27	4.92 ± 0.32	NS
ECOG PS (0/1/2)	162/35/5	141/61/9	<0.01
Child–Pugh score	5.83 ± 1.09	6.29 ± 1.35	<0.01
ALBI grade (1/2/3)	87/132/7	65/160/17	<0.01
ALBI score	−2.38 ± 0.47	−2.23 ± 0.53	<0.01
TNM stage (2/3/4)	25/69/116	30/63/134	NS
Within Up‐to‐7 criteria	94 (42%)	99 (34%)	NS
Treatment duration (days) (median)	118 (1–1932)	104 (1–1914)	NS
Grade ≥ 2 AEs	131 (58%)	119 (41%)	NS
Therapy after first line, TACE	76 (34%)		
Radiation therapy	23 (10%)		
Second line TKIs	85 (38%)		
ICI	50 (22%)		
Operation	4 (1.8%)		
Radiofrequency ablation	3 (1.3%)		

Values are *n*, *n* (%), or mean ± SD, unless otherwise specified.

AE, adverse event; AFP, alpha‐fetoprotein; ALBI, albumin‐bilirubin; APRI, AST to platelet ratio index; BMI, body mass index; DCP, des‐gamma‐carboxy pro‐ thrombin; ECOG PS; Eastern Cooperative Oncology Group performance status; FIB‐4, fibrosis‐4; ICI, immune checkpoint inhibitor; NS, not significant; TACE, transarterial chemoembolization; TKI, tyrosine kinase inhibitor; TNM, tumor‐node‐metastasis.

### Efficacy of post‐progression therapy after lenvatinib

**Figure 2:**
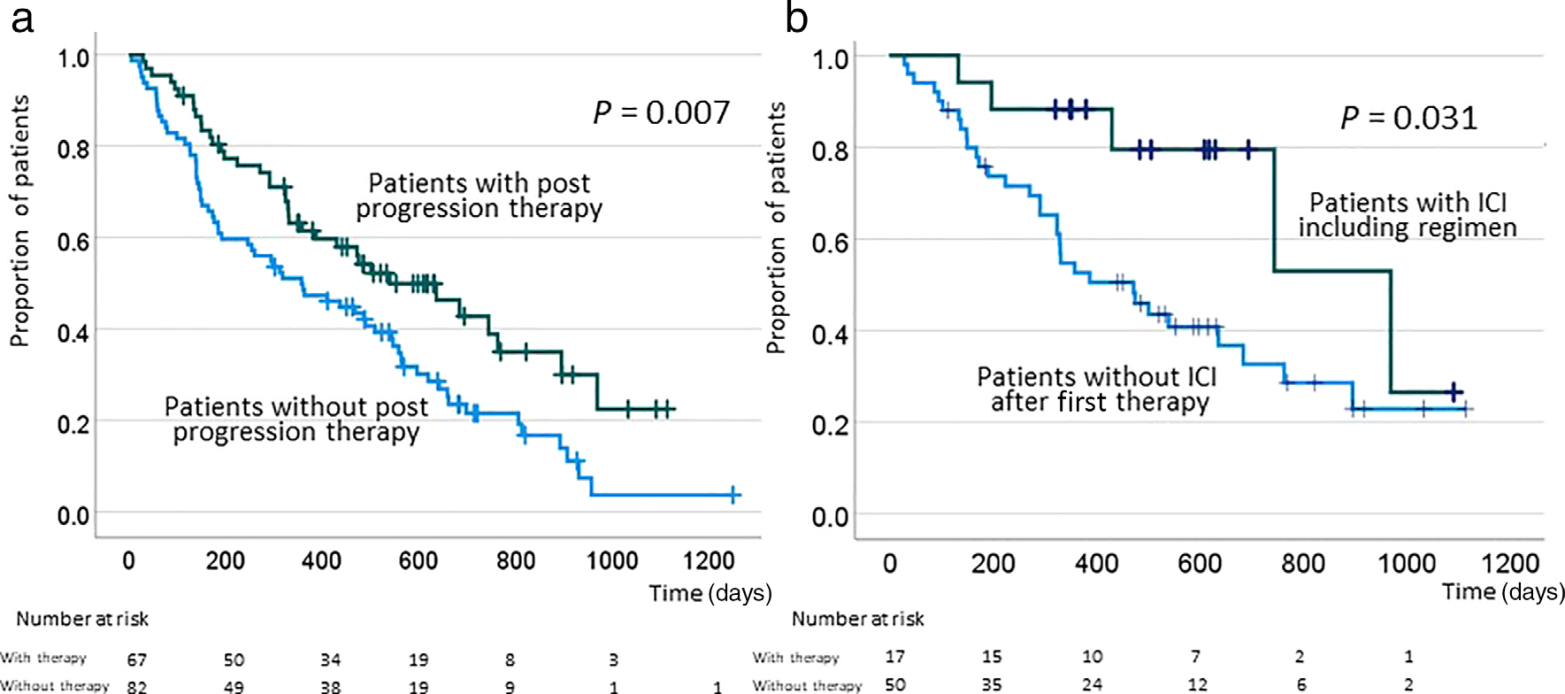
Overall survival in patients who received lenvatinib (a) according to the use of post‐progression therapy and (b) according to whether use of an immune checkpoint inhibitor (ICI) as post‐progression therapy compared with patients who received other therapies.

Although ICIs are often used as post‐progression therapy following lenvatinib, the preferred post‐progression therapy after lenvatinib has not yet been established. Therefore, we limited the analysis of post‐progression therapy to patients who received lenvatinib as first‐line therapy. In this analysis, we examined the outcomes of 204 patients treated with lenvatinib according to whether or not they received post‐progression therapy. Totally, 88 patients received post‐progression therapies, which included surgery in 2 patients, TACE in 30 patients, chemotherapy in 36 patients, and ICI‐based therapy in 24 patients (23 received atezolizumab and bevacizumab, and 1 received nivolumab and ipilimumab owing to participation in a clinical trial). Patients who received post‐progression therapy were divided into two groups: 24 patients with ICI‐included therapy, and 64 patients without ICI‐included therapy. The background characteristics of two groups are summarized in Table 5. As shown in the table, patients using ICI‐including therapy had significantly lower CP scores but no difference in the ALBI score. In addition, patients who used the ICI had fewer reliefs of side effects due to AEs in first‐line TKI treatment. On the other hand, patients with Grade 2 or higher AEs had low CP scores (5.67 ± 1.00 *vs* 6.15 ± 1.51, *P* < 0.001) and low ALBI scores (−2.42 ± 0.45 *vs* –2.37 ± 0.64, *P* = 0.028), indicating that they had good liver function. The log‐rank test revealed that OS was significantly longer in patients who received post‐progression therapy than in those who did not (Fig. 2a). Among the patients who received post‐progression therapy following lenvatinib, OS was significantly longer in those who received an included ICI therapy than in those who received other therapies, suggesting that ICI therapy is effective after lenvatinib (Fig. 2b).

**Table 5:** Characteristics of patients with and without immune checkpoint inhibitor including therapy after lenvatinib treatment

	With ICI including therapy	Without ICI including therapy	*P*‐value
*N*	24	64	
Age (years)	69.6 ± 9.7	70.7 ± 9.4	NS
Sex (male/female)	21/3	50/14	NS
BMI (kg/m^2^)	23.7 ± 3.6	23.9 ± 3.9	NS
Etiology (viral/non‐viral)	12/12	32/32	NS
Complication: hypertension	14 (58%)	45 (70%)	NS
Complication: diabetes	10 (42%)	18 (28%)	NS
APRI	1.05 ± 0.97	1.17 ± 1.04	0.031
FIB‐4 index	4.97 ± 0.27	4.92 ± 0.32	NS
Child–Pugh score	5.71 ± 1.12	5.84 ± 1.20	<0.01
ALBI score	−2.40 ± 0.56	−2.39 ± 0.50	NS
TNM stage (2/3/4)	7/7/10	10/25/29	NS
Within Up‐to‐7 criteria	10 (42%)	28 (44%)	NS
Treatment duration (days) (median)	180 (4–870)	105 (3–922)	NS
Change therapy by PD	18 (75%)	35 (55%)	NS
ORR	8 (33%)	15 (24%)	NS
DCR	17 (71%)	47 (73%)	NS
Change therapy by AE	5 (22%)	31 (48%)	0.019
Grade ≥ 2 AEs	13 (54%)	47 (73%)	NS
AE, HFS	2 (8%)	19 (30%)	0.018
AE, proteinuria	4 (17%)	10 (16%)	NS
AE, hypertension	6 (26%)	13 (20%)	NS
AE, fatigue	2 (9%)	10 (16%)	NS
AE, appetite loss	0 (0%)	6 (9%)	0.024
Other therapy, TACE	1 (4%)	32 (50%)	<0.01
Radiation therapy	1 (4%)	11 (17%)	0.04
Second line TKIs	4 (17%)	31 (48%)	<0.01
Operation	1 (4%)	1 (2%)	NS
Radiofrequency ablation	0 (0%)	3 (5%)	NS

Values are *n*, *n* (%), or mean ± SD, unless otherwise specified.

AE, adverse effect; APRI, AST‐to‐platelet ratio index; BMI, body mass index; DCR, disease control rate; FIB‐4, fibrosis‐4; ICI, immune checkpoint inhibitor; NS, not significant; ORR, overall response rate; TACE, transarterial chemoembolization; TKI, tyrosine kinase inhibitor; TNM, tumor‐node‐metastasis.

## Discussion

TKIs, including sorafenib and lenvatinib, have been used for many years as first‐line systemic therapy in patients with TACE‐refractory HCC. Recently, however, the combination of atezolizumab and bevacizumab was shown to be superior to sorafenib, and the combination is increasingly being used as the first‐line treatment for HCC.^7^, ^11^ However, no reports have described the efficacy of atezolizumab and bevacizumab as second‐line therapy following treatment with the TKIs sorafenib and lenvatinib as first‐line therapy. Therefore, in this study, we investigated the efficacy of post‐progression therapy in patients who received sorafenib and lenvatinib as initial therapy, and then assessed the efficacies of using an ICI for post‐progression therapy. We found that patients who received post‐progression therapy had a significant increase in OS, indicating favorable efficacy of treatment, compared with patients who did not receive post‐progression therapy. It important to maintain performance status (PS) and liver function in order to continue post‐progression therapy. Our study revealed that the liver function at the time of initial TKI therapy was better in patients who received post‐progression therapy than in those who did not. It seems likely that patients who were judged as having no response to treatment or who experienced AEs, which made continuation difficult, would be recommended to switch to the next treatment as soon as possible, as there were no significant differences in TKI efficacy or AEs between the groups of patients who received post‐progression therapy and those who did not.

In the REFLECT study, the median survival time was 13.6 months in the lenvatinib group and 12.3 months in the sorafenib group; our results were slightly lower than these.^3^ One reason for the difference in results is that the current study included CP B patients based on real‐world clinical practice; the mean survival time (MST) for CP A patients was 15.0 months, which was significantly longer than that for CP B patients (8.2 months). A post hoc analysis of the REFLECT trial revealed that 156 patients (32.6%) who received any anticancer medication (including sorafenib, fluorouracil, and cisplatin) after lenvatinib had better OS than the overall population of patients who received lenvatinib (20.8 *vs* 13.6 months).^12^ In addition, Hiraoka *et al*. reported that patients with good liver function have a higher likelihood of post‐progression therapy after lenvatinib, contributing to prolonged prognosis.^13^ Recent reports have suggested that conversion therapy, such as surgery or radiofrequency ablation, is also effective after lenvatinib and should be considered as a treatment option, if possible.^14^, ^15^, ^16^ Our study demonstrates the efficacy of ICIs for post‐progression therapy, providing support for their consideration as a treatment option in this setting. In this study, patients who used the ICI had fewer reliefs from side effects due to AEs in first‐line TKI treatment. This may be because patients who were relieved of side effects were able to switch to second‐line TKI treatment because TKIs treatment was considered as effective. Patients with Grade 2 or higher AEs had significantly better liver function, suggesting that these patients may have had more side effects due to less dose reduction and better medication compliance. In this study, patients who received a second‐line TKI after lenvatinib did not show a significant prolongation in OS compared to patients who did not receive systemic chemotherapy including ICI and TKI (data not shown). Currently, the efficacy of TKIs after lenvatinib treatment has not been established, and further studies are needed.

A limitation of this study is that there were no strict criteria for switching to second‐line treatment because the decision to change to the second‐line treatment or continue the first‐line treatment in case with progressive disease (PD) depended on the attending physician. In general, the second‐line therapy is usually started following the diagnosis of PD. However, before the availability of regorafenib in 2018, sorafenib was sometimes continued because there were no suitable second‐line therapy, even though the response to first‐line therapy was judged as PD. Second, the cancer stage at the start of TKI therapy was lower in the lenvatinib group than in the sorafenib group. Recent real‐world clinical studies and propensity score matching analysis of aHCC comparing lenvatinib and sorafenib have shown that lenvatinib treatment is more effective.^17^, ^18^, ^19^ In addition, the molecular‐targeted therapy is to start as early as possible for patients who are unsuitable for TACE, although chemotherapy has been formerly introduced only after repeated TACE.^11^ Changes in the position of chemotherapy for HCC over time may be a limitation to the present analysis.

In conclusion, post‐progression therapy after initial TKI treatment may prolong the prognosis of patients with HCC, and it should be strongly considered if possible. In particular, the use of an ICI as part of post‐progression therapy had a favorable effect on prognosis, and ICIs should be considered after initial TKI therapy.

### Patient consent

The need to collect informed consent from the patients was waived because this was a retrospective study. Information about this study was published in our institute, and patients could ask for their data to be withdrawn from the analysis.

## Supporting information


**Figure S1.** Kaplan–Meier plots of (a) overall survival and (b) progression‐free survival according to treatment with sorafenib and lenvatinib. Based on the propensity score matching analysis, factors including age, etiology, Child–Pugh score, frequency of extrahepatic metastasis, and frequency within Up‐to‐7 criteria were adjusted, and the analysis included 163 patients each for sorafenib and lenvatinib.

## Data Availability

All data generated or analyzed during this study are included in this published article.
