# Supplementary figures and images for: Significance of post‐progression therapy after tyrosine kinase inhibitors for advanced hepatocellular carcinoma
Source: JGH Open. 2022 May 25;6(6):427–33. doi: 10.1002/jgh3.12772 (PMC9218537; doi:10.1002/jgh3.12772)

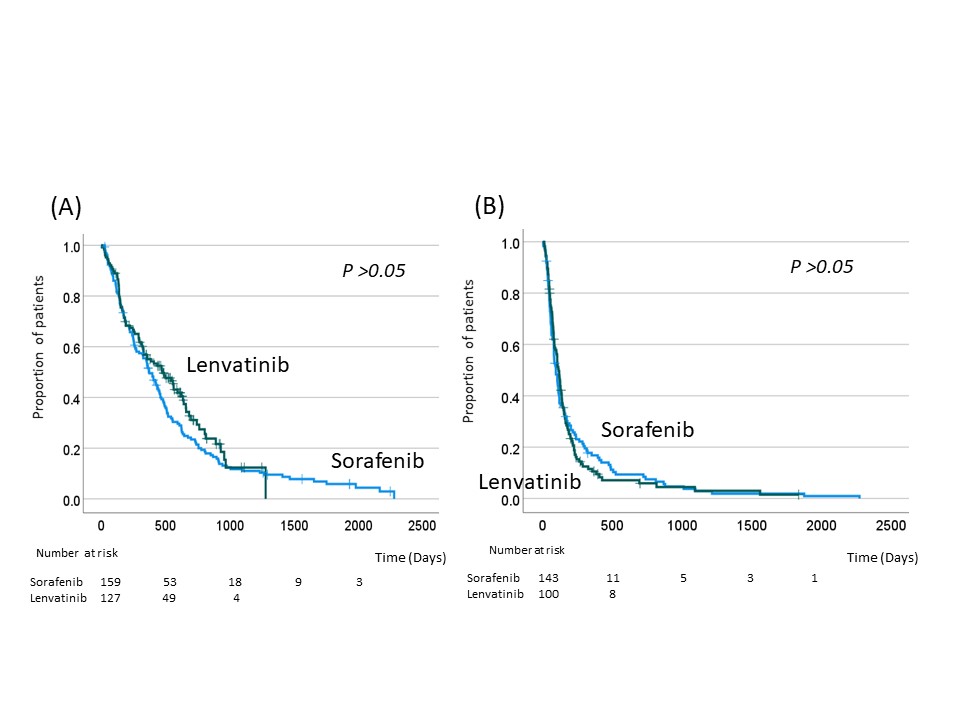

Supplement: Supplementary file 1 — Figure S1. Kaplan–Meier plots of (a) overall survival and (b) progression‐free survival according to treatment with sorafenib and lenvatinib. Based on the propensity score matching analysis, factors including age, etiology, Child–Pugh score, frequency of extrahepatic metastasis, and frequency within Up‐to‐7 criteria were adjusted, and the analysis included 163 patients each for sorafenib and lenvatinib. [file JGH3-6-427-s001.jfif]
